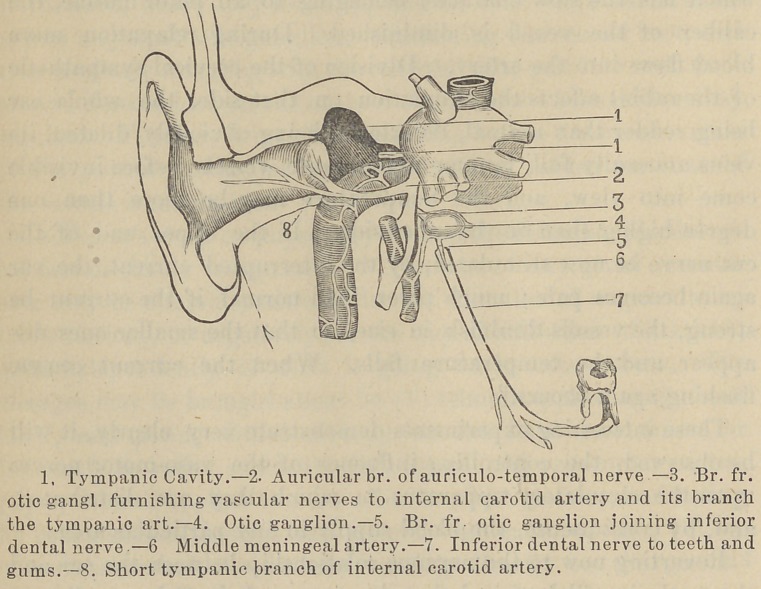# Correlated Diseases of the Teeth and Ear

**Published:** 1882-08

**Authors:** Joseph Richardson


### THE DENTAL REGISTER.

Vol. XXXVI.] AUGUST, 1882. [No. 8.

### Correlated Diseases of the Teeth and Ear.

BY JOSEPH RICHARDSON, M.D., D.D.S.

Read before the Indiana State Dental Association, June, 1882.

While there is, on the part of dentists, a general recognition of the existence of sympathetic nervous relations between the teeth, and other organs or regions more or less remote, it is to be feared that anything like a critical knowledge of the exact nature of this relationship is quite vague and limited. In so far at least as the subject under consideration is concerned, there is, it is to be apprehended, a pretty solid sub-stratum of truth in what Dr. Sexton, an eminent aural specialist of New York, has to say of us, namely, that "the apathy which has always existed on the part of the medical profession regarding the teeth has left the treatment of these organs in the hands of men who have occupied themselves almost exclusively with its mechanical department, and who, as a rule, have but little to do with the teeth in a medical aspect," and then laments, "that a field of such interest has been abandoned by the profession."

While we may wince a little under this accusation, it may be as well to accept it as at least a partial statement of the truth if it will only serve as a stimulus to higher effort in the direction of an increased knowledge on our part of the "medical aspects" of our calling. That we have made some progress, however, in the direction indicated, would appear from the fact that, in a prize essay on this subject published in the *American Medical Journal*, 1880, Dr. Sexton had recourse to dental authorities for a large part of the pathological data in relation to the diseases of the teeth and gums, which it was necessary for him to employ in elucidation of his subject. Besides, dentistry as a science is quite young—less than half a century old, while otological science may be said to date from the time of Du Verney's treatise on the ear, nearly two hundred years ago. It took the medical profession about two centuries to emerge from darkness into light in respect to the true nature of the nervous relationship we are considering. The writer already quoted says:

"The otology of the fathers, indeed, did not include a knowledge of a nervous relationship; and even at the beginning of the present century the writings of Saunders, Saissy, and others allude to diseases of the teeth as affecting the ears, in a manner most meager, although anomalies of the throat were spoken of as causing deafness; principally, however, as offering a mechanical obstruction to the faucial openings of the Eustachian tubes. Even Toynbee, Wilde, Triquet, and their contemporaries, failed to contribute to the knowledge of this subject in any material manner. The important work of clearing up this subject was left to to the otologists of the present day, and in turning to our principal writers, we find the nervous relationship of the teeth and ears clearly recognized." The evolution of knowledge was slow on this subject, but we accept the results with thanks, and should endeavor to turn them to some practical account in the practice of oral surgery.

Availing myself of the researches of some of these later writers on otological science, I shall confine myself to as brief a consideration as possible of the sympathetic relations of the teeth and gums and the ear, and incidentally, of the brain. Even a superficial survey of this limited field of inquiry, for it might be extended as well to the eye and other organs or regions, must impress every one with its practical value to us as practitioners of oral surgery, and I therefore solicit your patient attention to what may be offered.

It is not so much of mere functional disorders of the ear, manifesting themselves as reflex neuralgias, that I shall speak, but of structural lesions or trophic diseases affecting the several parts of the auditory apparatus. This aspect of the subject is one about which the greater interest centers considered in its relation to diseases of the teeth and gums as the primary source of such injuries through the operation of the the law of reflex action. It will be curious and instructive to know how deaf-mutism and fatal convulsions in childhood, or partial or complete loss of the sense of hearing in adult life may be induced primarily by the eruption of the deciduous teeth in the one case, or decayed teeth in the other, for all these grave sequences are sometimes clearly traceable to the primary disorders of the oral cavity. The relation of a single typical case will serve as a text to point out the nature of the relationship between diseases of the teeth and trophic tissue-changes of the ear.

Dr. Charles H. Burnett, of Philadelphia, author of a treatise on the ear, relates the following case. I shall omit much that relates to topical treatment of the affected organ.

"In July, 1878, Mrs. B. P., aged forty. consulted me in reference to a discharge from the right ear, which had annoyed her for sometime. Her statement was, that the discharge had set in after an attack of not severe ear-ache some months previous; that the discharge then grew slowly less, when another attack of pain in the ear was followed by an increased discharge, again to diminish and again to increase, after the now frequent attack of pain. The hearing had at no time been affected to any extent, being only slightly dulled sometimes, apparently by retention of small quantities of discharge. The latter had never been very great and the chief annoyance seemed to be the recurrence of pain and the odor of decomposing matter in the ear. There had been some ear disease on this side after scarlatina in childhood, but the ear had healed, and remained a good one until quite recently, when the above symptoms showed themselves. The patient had no reason to assign for her aural disease.

"Upon inspection, the membrana tympani was found perforated in the inferior posterior quadrant, at which point there were some small granulations.

"The posterior wall of the auditory canal, near the membrana tympani, was ulcerated, showing a granulating spot, and the fundus of the canal was bathed with light-colored pus. The Eustachian tube was easily inflated by Valsalva's method, and the perforation whistle readily obtained. The mucous membrane of the tympanic cavity, seen through the perforation after the ear was cleansed, appeared healthy, thus showing that the disease was confined chiefly to the outer surface of the membrana tympani and the posterior wall of the canal, in fact, it suggested itself to me that the disease might have originated in the canal and spread to the drumhead ; but it did not occur to me until after prolonged treatment, that the disease in the ear might be *purely reflex, and due to several* diseased teeth in the lower jaw on the right side, as it finally was shown to be."

The treatment of this case was continued for nearly four months before Dr. Burnett discovered the origin of the ear trouble. Partial relief was obtained at times, only to be followed by a recurrence of the unfavorable symptoms. At the patient's last visit, Dr. Burnett says: "Though the objective symptoms were good, the patient said she had had some of the old pain, which she had felt so often and knew so well as a forerunner of renewed ear discharge. A casual remark about her teeth, viz.: that they had long given her discomfort, led me to look into her mouth; on the right side of the lower maxilla, the *first and second molars were seen to be largely decayed and the gums about them inflamed and sensitive.* This at once furnished a clue to the origin and stubborness of the disease in the ear. As soon as these teeth were extracted, all discomfort in the mouth ceased, and from that *day to this, two years, no pain or any other symptom of aural disease has shown itself.*"

There is, I think, no mistaking the testimony of this case. The prompt subsidence of the aural trouble following the extraction of the teeth points clearly to the sympathetic and dependent character of the former. A study of the nervous relationship between the teeth and gums, and the external meatus, or auditory canal, the part most prominently affected in the related case, and the manner in which trophic lesion of the latter is established by reason of such relationship, will serve as a key to an understanding of the source and nature of similar morbid processes sometimes affecting other parts of the auditory apparatus, as the Eustachian tube, the middle ear or tympanic cavity, and the labyrinth.

Before tracing the continuity of nerve fiber between the teeth and ear, there are some ascertained facts relating to nerve function, a knowledge of which seems essential to an intelligent comprehension of the subject under treatment.

Dr. Edward Woakes, of London, one of the most careful and intelligent exponents of the doctrine of reflex diseases of the nervous system, after relating the case of an enlarged gland below the external ear, associated with disease of the external meatus, and cured by the extraction of a decayed tooth, says: "Now the question before us is to find the solution of the train of symptoms just detailed. We have the phenomena of pain, inflammation, and suppuration, occurring in an organ widely separated from the recognized exciting cause, and in tissues histologically distinct from those manifesting the morbid changes. The only obvious connecting link between the regions interested is the continuity of nerve fibre. The simple continuity of sensori-motor nerves is insufficient to produce the conditions under review, we must seek yet farther for the true medium by which they are brought about. This will be found in the relations of the vaso motor nerves, and the functions which it is their office to fulfill.

"As it is this portion of the nervous system, that is mainly concerned in the morbid processes we are examining, it will be necessary briefly to enumerate the chief points in its economy. By far the most important fact in connection with the vaso-motor system is that, with one or two exceptions, all sensori-motor nerves comprise fibres belonging to it, and that these fibres run in a contrary direction to the cerebro-spinal nerve with which they are associated. Thus in speaking of a cerebro-spinal nerve, say the vagus, we describe it as pursuing a course *from* the medulla to the respiratory organs, and the several viscera which it supplies. At the same time it must be remembered that it contains other fibrillæ in its sheath running *from* the viscera towards the nerve centre, some of which at intervals leave the sheath and enter a ganglion of the sympathetic, in their course to the general vaso-motor center, situated in the medulla oblongata, at a point which has scarcely been determined for the human subject, though it has been accurately fixed in the rabbit. These fibers then are centripetal or afferent in their function, conveying impressions from the tissues to the sub-centers constituted by the ganglion, or to the general vaso-motor center.

When these fibers enter a ganglion they communicate with its caudate cells, which important fact brings them into communication with other nerves coming from different directions, to the same ganglion; when the afferent fibers leave the ganglion, they pass backwards by one of the two cords leaving the ganglion to join the spinal cord, and in it traverse the anterior columns of the cord in an upward direction to reach the primary vaso-motor center.

The *efferent* or *centrifugal* fibers in reflex relationship with the foregoing, or *afferent* fibers, follow an exactly similar course from the general center *downwards*, likewise along the anterior columns of the cord, which they leave opposite an inter-vertebral foramen to join a sympathetic ganglion, constituting its second root, and after similarly mingling with the caudate cells, quit it to seek their several destinations on the coats of the arteries whose caliber they regulate. Further, it is to be noted that, by the automatic action of the *general* vaso-motor center, the normal caliber or tone of the vessels is maintained."

In Foster's work on physiology we find the following physiological fact distinctly recognized: "The tone of any given vascular area may be altered, positively in the direction of augmentation (constriction), or negatively, in the way of inhibition (dilitation), quite independently of what is going on in other areas. The changes may be brought about by (1) stimuli applied to the spot itself, and acting either directly on the local mechanism, or indirectly by reflex action through the general vaso-motor center; (2) by stimuli applied to some other sentient surfaces, and acting by reflex action through the general vaso-motor center; (3) by stimuli (chemical, blood-stimuli) acting directly on the general vaso-motor center."

In the cases under consideration, the change in the tone of the arteries distributed to the ear would be induced by the class of stimuli spoken of by Foster under the second head, or by stimuli applied to sentient surfaces (the teeth and gums).

The physiological action of the vaso-motor system upon the tonicity or caliber of the arteries is thus demonstrated by experimental tests. I quote from Foster's Physiology: "Nerve fibres belonging to the sympathetic system are distributed largely to the blood vessels, but their terminations have not as yet been clearly made out (we may say parenthetically that by "blood vessels" is here meant the arteries chiefly if not altogether, as it is the function of the vaso-motor system to regulate the caliber of the vessels to which they are distributed, and as this can only be done by their action on muscular tissue, their action is limited to arteries, since the coats of the capillaries are destitute of muscular fiber, and the veins have but very slight traces of it). By galvanic or mechanical stimulation, this muscular coat may in the living artery be made to contract. During this contraction which has the slow character belonging to all plain muscle, the caliber of the vessel is diminished. During relaxation more blood flows into the artery. Division of the cervical sympathetic of the rabbit affects the circulation on that side, the whole ear being redder than normal, its arteries being obviously dilated, its veins unusually full, innumerable minute vessels before invisible come into view, and the temperature may be more than one degree higher than on the other side. If the upper end of the cut nerve be now stimulated, by the interrupted current, the ear again becomes pale; much paler than normal if the current be strong, the vessels diminish in size, so that the smaller ones disappear, and the temperature falls. When the current ceases, flushing again occurs."

These interesting experiments demonstrate very clearly, it will be observed, the controlling influence of the vaso-motor nerves upon the circulatory apparatus to which they are distributed, and, by consequence, the blood supply in any particular area.

Reverting now to the nervous relationship between the ear and the teeth, it will be found that, by means of the Otic ganglion, a nervous connection is established in the following manner: "The nerves supplying the mucous membrane of the tympanic cavity, as well as that of the Eustachian tube and Mastoid cells, are derived from the tympanic plexus, an anastomasis between the *Otic ganglion*, petrosal ganglion of the glosso-pharyngial nerve, and the carotid plexus, by means of the superior cervical ganglion of the sympathetic. Now, by means of the Otic ganglion, the soft palate, the drum-head, and the tensor tympani muscle, the lining mucous membrane of the cavity of the drum, and the integument of the external ear, are put into sympathetic relation with each other and other parts of the nervous system."* In studying this nervous connection, it is needful to bear in mind the important and essential part the Otic ganglion plays as a *medium* of communication between the teeth and the ears. This ganglion is situated on the inner side of the sensory division of the inferior dental nerve, and communicates with it by two or three small branches, and also with the tympanic plexus, of which it is a component. (See accompaning diagram.)

1, Tympanic Cavity.—2. Auricular br. of auriculo-temporal nerve.—3. Br. fr. otic gangl. furnishing vascular nerves to internal carotid artery and its branch the tympanic art.—4. Otic ganglion.—5. Br. fr. otic ganglion joining inferior dental nerve.—6 Middle meningeal artery.—7. Inferior dental nerve to teeth and gums.—8. Short tympanic branch of internal carotid artery.

*Burnett.

Applying the facts in connection with the continuity of nerve fiber between the teeth and the ear, and the functions of the vaso-motor system, it remains to explain the *modus operandi* by which irritation in a diseased tooth or the gums may give rise to trophic lesions in the ear.

For purposes of illustration, I will take the case related by Dr. Burnett, where perforation of the tympanum and ulceration of the external auditory canal existed, and which, after persistent topical treatment had failed, was speedily and permanently cured by extraction of the decayed molars on the same side.

It has been shown, in tracing the nervous connection between the teeth and the ear, that the region embracing the tympanic cavity, the drum-head, and external meatus, constituted an area correlated to the point of irritation in the diseased teeth by means of the Otic ganglion. This ganglion is connected with the plexus of the sympathetic distributed to and over the external carotid artery, the branches of the latter supplying the external auditory canal with blood. It is easily seen, therefore, how this part of the ear becomes an area correlated to the point of irritation in the diseased teeth, through the medium of the Otic ganglion. Now, according to Dr. Burnett, as the effect of any irritation in a vaso-motor nerve-tract is to excite vascular dilitation within the area correlated to such point of irritation through diminished inhibitory nerve-power, we have following this impaired tonicity of the arteries, first, dilitation, then hyperemia or congestion, inflammation, pain, effusions, suppuration, and ulcerative destruction of tissue, as the case may be.

The same condition of altered blood supply may, from the same cause, occur in the drum-head, through the connection existing between the inferior dental nerve, the Otic ganglion, and the internal carotid plexus of the sympathetic, in which case the blood supply of the tympanum through its branch of the internal carotid is augmented through a loss of inhibitory power in the vessel. In like manner, trophic disease of the middle ear, or tympanic cavity may ensue by reason of altered blood-supply induced by an irritation conveyed over a nervous area, the components of which are the tympanic nerve or plexus on one side, the Otic ganglion as a medium, and the inferior dental nerve on the other side.

This paper would be incomplete without some reference to the correlated diseases of the gums and the ear during the period of teething. From what has been already written, it will not be difficult to understand the sources of danger during this process. There is something startling and almost pathetic in the statistical revelation of the sufferings, disabilities, and mortality accompanying the eruption of the deciduous teeth. Out of 80 infants under fourteen months, examined by Dr. Wreden, of St. Petersburg, more than 80 per cent. had some form of ear affection. As these ear diseases were coincident with the period of teething, it is presumable that they were associated, in a large degree at least, with the latter process.

Respecting reflex diseases of the ear during infancy, Dr. Woakes justly and forcibly remarks: "The field of inquiry thus opened possesses interests far beyond those which appertain to the ear as an organ of special sense. Even from this narrow point of view, the possible loss of hearing as the result of destructive processes in the ear occurring as complications of infantile ailments, the issue is one of great anxiety. For a child who thus becomes deaf before it has learned to talk will be dumb also, producing the pitiable object of an intelligent being deprived of two channels of communication with the outside world.

"As already intimated, it is on the very threshold of life that these sympathetic ear symptoms are brought into prominence. A child is cutting its teeth, and while the gums are yet swollen it suffers acutely from ear-ache. How do we know this, seeing the child can not speak? Any one accustomed to watch carefully the symptoms of these little patients will scarcely fail to discern in the troubled face, the resting of the head on the nurse, the thrill of agony which passes across its features accompanied with piteous cries or shrieks when its position is moved, especially if this be done suddenly, and more than all, the constant raising of the little hand to the side of the head; no one who has watched these symptoms will fail to connect them with the most agonizing of all the sufferings of early life—*ear-ache.*

"Now, the point I wish to emphasize is this: The pain thus experienced is not what we vaguely call neuralgia; it is a definite trophic change, an inflammation taking place in the deeper seated tissues of the ear, beginning with congestion and stretching of an acutely sensitive region, passing on to exudation and suppuration, and capable of being recognized, if the proper means are used for doing this. If the case be seen early all these symptoms are at once removed by a free incision of the swollen gums. But it often happens that those trophic changes just alluded to have set in before the practitioner is called upon to see his patient. The gums are, however, duly lanced, and very properly so, because reflex irritation is thereby lessened. But, to the disappointment of practitioner and parents, the little patient is not cured. Then commences the orthodox role of treatment. Cold to the head, hot baths, mustard plasters, and perhaps a calomel purge, followed by enemata. Still the patient gets worse, convulsions set in, and the child dies."

Thus, though the original irritation leading to these results may no longer exist by reason of the lancing of the gums, yet a veritable otorrhœa is established and perpetuated, menacing continually the life of the child by an extension of the inflammatory processes to the brain, an occurrence for which every facility is arranged by the intimate communications, which in the infant especially, exist for such an issue.

Aside from the dangers resulting in these cases from the pressure of effused muco-purulent fluid within the tympanic cavity, and the pain consequent thereon, which threaten the life of the child by the irritation excited in the cerebral centers, there is a particular structural arrangement which greatly favors the occurrence of fatal convulsions in the infant. At this early age the petrous and squamous portions of the temporal bone are developed separately, and between them exists a well-marked fissure. At this fissure the dura-mater dips down into the cavity of the tympanum or middle ear, becoming continuous with its muco-periosteal lining. This process of dura-mater carries with it a rich endowment of vessels derived from the middle meningeal artery, and which are the vessels belonging to the cavity. In the progress towards adult life this fissure becomes more or less obliterated, though the vascular connection with the arteries remains.

In the light of these facts it would seem useless to attempt to impress the gravity of the perils which environ the infant by reason of the reflex structural lesions so frequently occurring primarily from the irritation associated with teething. They carry their lesson with them, and point to timely and judicious treatment of the gums during this critical period.

On a review of the subject of this paper, which I am conscious has been imperfectly, and, I am sure, by no means exhaustively, treated, a question of peculiar interest to the dentist presents itself for consideration. If irritation having its source in the various structures of the mouth is competent to produce trophic diseases of the ear in the manner related, may not irritation proceeding from morbid conditions of the ear, and other organs give rise to trophic diseases of the several structures of the oral cavity. And if this be true, may not a better knowledge and a more general recognition of these reflex nervous relationships furnish the key to a solution of the origin of many affections of the buccal cavity the etiology of which is now unknown or obscure. I earnestly commend this aspect of the subject to the thoughtful and intelligent consideration of the profession.

## Figures and Tables

**Figure f1:**